# Genome-Wide Association Study and Phenotype Prediction of Reproductive Traits in Large White Pigs

**DOI:** 10.3390/ani14233348

**Published:** 2024-11-21

**Authors:** Hao Zhang, Shiqian Bao, Xiaona Zhao, Yangfan Bai, Yangcheng Lv, Pengfei Gao, Fuzhong Li, Wuping Zhang

**Affiliations:** School of Software Technology, Shanxi Agricultural University, Jinzhong 030801, China; zhanghao09_32@163.com (H.Z.); baoshiqian2024@163.com (S.B.); 18536497330@163.com (X.Z.); m747241764@163.com (Y.B.); l1978453400@163.com (Y.L.); gpf800411@126.com (P.G.)

**Keywords:** large white pigs, reproductive traits, genome-wide association study, genomic selection, machine learning

## Abstract

In a study involving 385 Large White pigs, a genome-wide association study (GWAS) was conducted to investigate reproductive traits, specifically the number of healthy litters (NHs) and the number of weaned litters (NWs). Several SNP loci, including ALGA0098819, ALGA0037969, and H3GA0032302, were significantly associated with these traits. In the combined-parity analysis, candidate genes, such as *BLVRA*, *STK17A*, *PSMA2*, and *C7orf25*, were identified. GO and KEGG pathway enrichment analyses revealed that these genes are involved in key biological processes, including organic synthesis, the regulation of sperm activity, spermatogenesis, and meiosis. In the by-parity analysis, the *PLCXD3* gene was significantly associated with the NW trait in the second and fourth parities, while *RNASEH1*, *PYM1*, and *SEPTIN9* were linked to cell proliferation, DNA repair, and metabolism, suggesting their potential role in regulating reproductive traits. These findings provide new molecular markers for the genetic study of reproductive traits in Large White pigs. For the phenotypic prediction of NH and NW traits, several machine learning models (GBDT, RF, LightGBM, and Adaboost.R2), as well as traditional models (GBLUP, BRR, and BL), were evaluated using SNP data in varying proportions. After PCA processing, the GBDT model achieved the highest PCC for NH (0.141), while LightGBM reached the highest PCC for NW (0.146). The MAE, MSE, and RMSE results showed that the traditional models exhibited stable error rates, while the machine learning models performed comparatively better across the different SNP ratios. Overall, PCA processing provided some improvement in the predictive performance of all of the models, though the overall increase in accuracy was limited.

## Simple Summary

Pig farming is a vital pillar of Chinese agriculture; however, the low heritability of sow fertility traits presents challenges to enhancing economic performance through traditional breeding methods. This study demonstrated that SNP markers associated with reproductive traits can be effectively identified through genome-wide association studies (GWASs), while a gene enrichment analysis revealed potential biological processes and signaling pathways related to these traits. Machine learning methods were applied to the phenotypic prediction of two key reproductive traits in Large White pigs, assessing their potential to improve prediction accuracy, selection efficiency, and breeding effectiveness.

## 1. Introduction

As a key pillar industry in China’s agricultural development, pig farming holds a dominant position in consumer meat consumption. The reproductive ability of sows decisively impacts the economic efficiency of pig farming, which is directly related to the productivity and economic returns of pig farms. Reproductive traits are estimated to have heritabilities ranging from 0.06 to 0.20 due to multiple factors, such as genetics, environment, and nutrition. Traditional hybridization and natural selection methods are slow in the improvement process for these low-heritability traits [1].

Genome-wide association studies (GWASs) are effectively used to explore genome–phenotype associations and identify causal loci or candidate genes. They have been widely applied in selecting and breeding reproductive traits in pigs [2]. For example, the GWAS method identified key loci for social influence traits in Yorkshire pigs and pinpointed the candidate gene *MT3*, which has both direct and social genetic effects on daily feed intake [3]. These findings are crucial for understanding the genetic mechanisms of reproductive traits. Additionally, the number of teats in sows significantly affects the survival rate of weaned piglets. A GWAS analysis of the number of teats in Canadian-lineage Large White pigs identified 21 significantly associated SNPs and a series of molecular markers and candidate genes located on chromosome 7, further enriching the genetic basis of reproductive traits [4]. The study by Sell-Kubiak et al. [5] identified 10 SNPs associated with the number of piglets at birth through GWASs, further revealing the genetic diversity of reproductive traits in Large White pigs. Zhang et al. [6] revealed the genetic mechanisms of reproductive traits in Duroc populations through GWASs and identified seven SNPs associated with the number of weaned piglets. The most significant SNP was located on *SSC17*, with additional potential SNPs on *SSC4* and *SSC10*. Conversely, Wang et al. [7] identified 13 genes, including *ATP5O*, *GHRHR*, and *TRIM55*, as key candidate genes, influencing 10 growth traits in four-way hybrid pigs.

With advancements in genomics research, genomic selection (GS) methodology has emerged as a means to improve traits with low heritability. GS uses a high density of Single Nucleotide Polymorphism (SNP) markers across the entire genome to estimate individual breeding values. The first application of GS was in dairy cattle, and today, the genetic gain for annual yield traits in Holstein cows in the USA has increased from about 50% to 100%. GS is now widely recognized and successfully applied in plant and animal breeding programs [8,9,10]. The accuracy of GS is affected by several factors, including the method of analysis for genomic prediction, reference population size, marker density, and heritability [11]. One key factor is the statistical model used to predict the breeding values of candidate individuals. Numerous researchers have worked on improving existing models and developing new ones to enhance the predictive power of GS [1,12,13,14]. Currently, the most commonly used methods for livestock and poultry genomic selection are parametric, primarily including the Genomic Best Linear Unbiased Prediction (GBLUP) [15], Bayes Ridge Regression (BRR) [16], and Bayes Lasso Regression (BL) [17] models. These linear models typically consider additive inheritance and ignore the complex nonlinear relationships that may exist between markers and phenotypes (e.g., epistasis, dominance, or genotype–environment interactions). It has been shown that considering nonlinearity may enhance the genomic prediction ability of complex traits [1].

Machine learning (ML) methods can capture implicit relationships between genotypes and phenotypes when handling high-dimensional genomic data. Methods such as Random Forest (RF), Gradient Boosting Decision Tree (GBDT), Light Gradient Boosting Machine (LightGBM) and Adaptive Boosting for Regression (Adaboost.R2) have demonstrated superior performance in genomic selection for reproductive traits [18]. Wang et al. [1] compared the genomic selection accuracy of the GBLUP and Bayes methods with machine learning methods, such as Support Vector Machine (SVR), Kernel Ridge Regression (KRR), Random Forest (RF), and AdaBoost.R2, on the reproductive traits of Large White pigs, demonstrating the superiority of the ML methods. Ornella et al. [18] compared the performance of Support Vector Regression, Random Forest, Reproducing Kernel Hilbert Space (RKHS), Ridge Regression, and Bayesian Lasso for genomic prediction on maize and wheat datasets with various trait–environment combinations and found that RKHS and Random Forest performed optimally. Additionally, each ML method has unique advantages and exhibits varying performances across different species and traits. Therefore, selecting the most suitable ML method for various species and traits is an important challenge.

In this study, we combined genome-wide association studies (GWASs) and phenotypic prediction models to identify single-nucleotide polymorphism (SNP) markers that significantly affect healthy litter size and weaning head number traits in a growing pig population and further explored relevant candidate genes. Additionally, a gene ontology (GO) enrichment analysis, a Kyoto Encyclopedia of Genes and Genomes (KEGG) pathway analysis [19], and a functional annotation of candidate genes were conducted to gain a deeper understanding of the mechanisms of action and the biological pathways of these genes in the organism. The genetic basis of reproductive traits (the number of healthy piglets and the number of weaned heads) in Large White pigs was systematically revealed. Using machine learning methods, we conducted phenotypic predictions for two key reproductive traits in Large White pigs to assess their potential for improving prediction accuracy, selection efficiency, and breeding effectiveness.

## 2. Materials and Methods

### 2.1. Data Source and Processing

**Figure 1:**
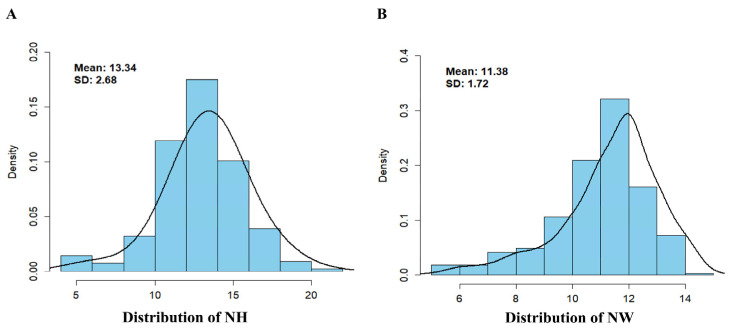
Phenotypic frequency distribution of two traits. (**A**) Distribution of the NH trait, showing the mean and standard deviation values. (**B**) Distribution of the NW trait, showing the mean and standard deviation values.

This study focused on 385 adult Large White pigs, raised by Shanxi Kaiyong Breeding Co., Ltd. (Jincheng, China), and records their reproductive data from March 2019 to August 2020, including the number of healthy litters (NHs) and weaned litters (NWs). Here, the number of healthy litters refers to the litters with a birth weight of greater than 1 kg. All individuals were fed a uniform standard diet. A descriptive statistical analysis was conducted on the phenotypic data (Table 1), and all the reproductive trait data of the sows met the standard of “mean ± 3 standard deviations” and followed a normal distribution (Figure 1). The generalized linear model (GLM) was also used to remove environmental factors such as year effect, season effect, strain effect, and sex effect, which corrected for these potential interfering factors and ensured that the phenotypic data better reflected the role of genetic factors. On this basis, all of the phenotypic data were standardized to further reduce the interference of the variation of environmental factors on the results [20].

(1) $$y_{ijklm}=\mu +Farm_{i}+Year_{j}+Season_{k}+Breed_{l}+Sex_{m}+e_{ijklm}$$

where $y_{ijklm}$ is the corrected phenotypic value, μ is the overall mean, $Year_{j}$ is the year of birth of the individual, $Season_{k}$ is the season of the birth of the individual, $Breed_{l}$ is its individual strain, $Sex_{m}$ is the sex of the individual, and $e_{ijklm}$ is the random residual.

**Figure 2:**
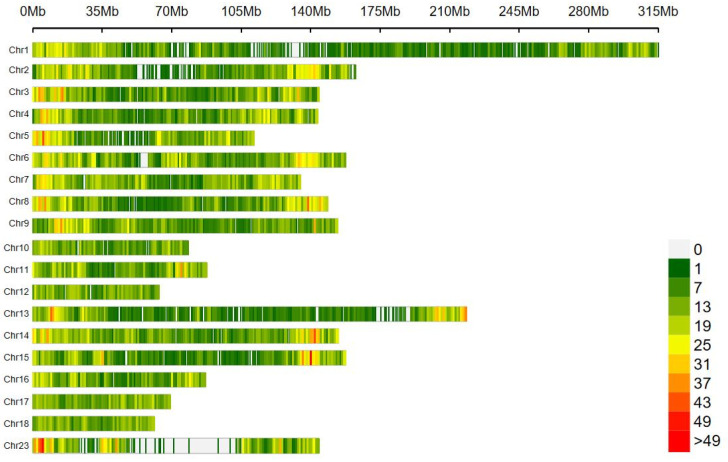
Distribution of genome-wide SNPs across chromosomes.

Genotypic data were quality controlled using PLINK v1.9 software [21]. The criteria included excluding individuals with an SNP deletion rate of greater than 2%, loci with a deletion rate of higher than 1%, SNP loci with a minimum allele frequency (MAF) of less than 3%, SNP loci that did not conform to the Hardy–Weinberg equilibrium, and SNP loci without a chromosomal location, ultimately retaining 35,795 valid SNPs. The genotype imputation for the undetected SNPs was performed using Beagle 5.2 software [22]. Different colors were used to indicate the number of SNPs contained within 1Mb, showing that the distribution of SNP loci on each chromosome was relatively uniform (Figure 2), which can be used for a subsequent association analysis.

### 2.2. Genome-Wide Association Analysis

This study utilized the generalized linear model in PLINK v1.9 software [18] to perform genome-wide association studies (GWASs) on both combined-parity data and by-parity data (first, second, third, and fourth parities) separately, and the parities and the calculated first five principal component analysis (PCA) results were added to the model as fixed effects to correct for potential population structural influences and to reduce the interference of environmental factors. The association analysis model used is as follows:

(2) y=Xb+Zμ+e

where y denotes the phenotype vector of reproductive traits after correction and standardization, X is the fixed effects matrix, Z is the random effects matrix, b is the fixed effects vector, μ is the random effects, and e denotes the residuals.

After completing the GWAS analysis, the results were visualized using the CMplot package (4.5.1) in RStudio. In this study, the threshold was adjusted to le-4 based on the Manhattan plot results, under which candidate genes were identified based on their physical location and function based on the *Sus-scrofa* 11.1 (http://www.ensembl.org/ (accessed on 2 October 2024)) reference genome from the Ensembl (https://useast.ensembl.org/index.html (accessed on 2 October 2024)) database for each potential SNP or the closest annotated gene identified as a candidate gene. Using the clusterProfiler package (4.10.1) [23], we conducted a gene function enrichment analysis on the annotated candidate genes based on the KEGG and GO databases [17] to further understand their biological functions. In the combined-parity analysis, we applied a strategy of selecting candidate genes within a 1 Mb range upstream and downstream of each SNP; whereas in the by-parity analysis, we annotated the gene closest to each SNP. Significant entries were identified based on the enrichment analysis results and relevant reports, allowing for the further identification of candidate genes for reproductive traits in Large White pigs.

### 2.3. Phenotype Prediction Model

#### 2.3.1. Conventional Model

In this study, three conventional models—Genomic Best Linear Unbiased Prediction (GBLUP), Bayesian Ridge Regression (BRR), and Bayesian Lasso Regression (BL)—were utilized for the phenotypic prediction of two traits. The GBLUP method, proposed by VanRaden and Habier et al. [15], is an effective approach for predicting the genotype values of target groups using genome-wide marker information. It constructs a genomic relationship matrix instead of a molecular relationship matrix. The GBLUP model is as follows:

(3) $$y_{c}=Xb+Zg+e$$

where $y_{c}$ is the corrected phenotypic value, X is the association matrix of the fixed effects with each individual, b is the fixed effect vector, Z is the association matrix, e is the residual vector, g is the additive random effect, obeying $g\sim N(0,G\delta_{\alpha }^{2})$, (G is the genomic relationship matrix of the additive effect, and $\delta_{\alpha }^{2}$ is the variance of the additive effect); the genomic relationship matrix G can be implemented by the following methods:

(4) $$G=\frac{WW^{\prime }}{2\sum_{j=1}^{m}p_{i}(1-p_{i})}$$

where W is the matrix of m×n genetic markers, m is the number of genetic markers, n is the number of individuals, and $p_{i}$ denotes the minor allele frequency of the i-th marker.

Two Bayesian regression methods—Bayesian Lasso Regression and Bayesian Ridge Regression —were used in this study. The Bayesian method consists of three essential components: prior, likelihood, and posterior. The prior probability is a quantitative measure that represents the parameters before the data analysis, and these parameters typically have their own prior distribution. The likelihood represents the conditional probability, and the posterior probability is derived by combining the prior and the likelihood using Bayesian theory.

#### 2.3.2. Machine Learning Model

In this study, four machine learning regression methods—Gradient Boosted Decision Tree (GBDT), Random Forest (RF), Light Gradient Boosting Machine (LightGBM), and Adaptive Boosting for Regression (Adaboost.R2)—were used to predict the phenotypes of two traits. GBDT is an iterative decision tree algorithm that can be viewed as an additive model consisting of m trees. At each step of the iteration, it constructs a learner that can reduce the loss along the direction of the steepest gradient to compensate for the shortcomings of the existing model and learns greedily, using a forward distribution algorithm during the training process. The results of multiple decision trees are then accumulated as the final predicted output by constructing a weak set of learners. The algorithm effectively combines the decision tree and integration ideas [14]. Random Forest is a machine learning algorithm developed by Breiman, which randomly selects different subsets from the provided data and uses them to build multiple different decision trees. It trains the sample data by combining multiple weak regressors, and the final predictions are obtained by voting or averaging, which makes the results of the overall model have a high degree of accuracy and generalization performance. Due to the “randomness of samples” and the “combination of decision trees”, Random Forest also has a better ability to resist overfitting [24]. LightGBM is an improved algorithm of GBDT with better computational efficiency and a better ability to deal with high-dimensional data. LightGBM has better computational efficiency and lower memory usage, and in the face of high-dimensional data, the LightGBM algorithm possesses better overfitting properties, which makes it more suitable for the pre-exploratory modeling of today’s increasing amount of modeling data [25,26]. Adaboost.R2 is an extension of Adaboost.R and Adaboost.M2, created to handle regression problems, which iteratively uses regression trees as weak learners, then increases the weight of incorrectly predicted samples and decreases the weight of correctly predicted samples. It creates a “committee” by integrating multiple weak learners [27].

In this study, the Bayesian optimization method [28] was used to optimize the model’s hyperparameters. By constructing a proxy model and leveraging previous parameter selection results, the method selects the parameter combination that is most likely to improve performance. This approach allows for finding a parameter combination that is close to optimal with fewer sampling iterations. This method effectively avoids local optima and tends towards global optima.

#### 2.3.3. Evaluation Indicators

The experiment was conducted using an AMD Ryzen 7 5800 processor (Advanced Micro Devices, Inc. (AMD), Santa Clara, CA, USA), NVIDIA GeForce RTX 3060 graphics card, 16 GB of RAM, and 512 GB of storage capacity. For the three ML methods, genotype data (coded as 0, 1, and 2) were used as input variables, and corrected phenotypes were used as response variables. The three ML methods were implemented using the Scikit-learn package [29], while GBLUP, BL, and BRR were implemented using the BGLR package (1.1.2) [30] for phenotype prediction.

A ten-fold cross-validation randomization process was used to calculate Pearson’s correlation coefficient (PCC), the mean square error (MSE), the mean absolute error (MAE), and the root mean square error (RMSE) between the predicted and the true phenotypic values to estimate the accuracy of the genomic prediction. Using the ten-fold cross-validation method, 385 individuals were randomly divided into 10 mutually exclusive subsets of similar sizes. Each time, 9 subsets were selected as the training set, and the remaining 1 subset was used as the test set. After completing one round, the process was repeated with 9 different subsets that were selected as the training set, and the results from all ten rounds were averaged. This cyclic training and validation process evaluated the model’s generalization ability and helped prevent overfitting.

(5) $$PCC_{\left(y,y_{pre}\right)}=\frac{\sum_{i=1}^{N}\left(y_{i}-\bar{y}\right)(y_{i,pre}-{\bar{y}}_{pre})}{\sqrt{\sum_{i=1}^{N}{\left(y_{i}-\bar{y}\right)}^{2}}\sqrt{\sum_{i=1}^{N}{\left(y_{i,pre}-{\bar{y}}_{pre}\right)}^{2}}}$$

(6) $$MSE_{\left(y,y_{pre}\right)}=\frac{1}{N}\sum_{i=1}^{N}{\left(y_{i}-y_{i,pre}\right)}^{2}$$

(7) $$MAE_{\left(y,y_{pre}\right)}=\frac{1}{N}\sum_{i=1}^{N}\left[|y_{i}-y_{i,pre}|\right]$$

(8) $$RMSE_{\left(y,y_{pre}\right)}=\sqrt{\frac{1}{N}\sum_{i=1}^{N}{\left(y_{i}-y_{i,pre}\right)}^{2}}$$

## 3. Results

### 3.1. Significant SNP Markers and Candidate Gene Identification

**Figure 3:**
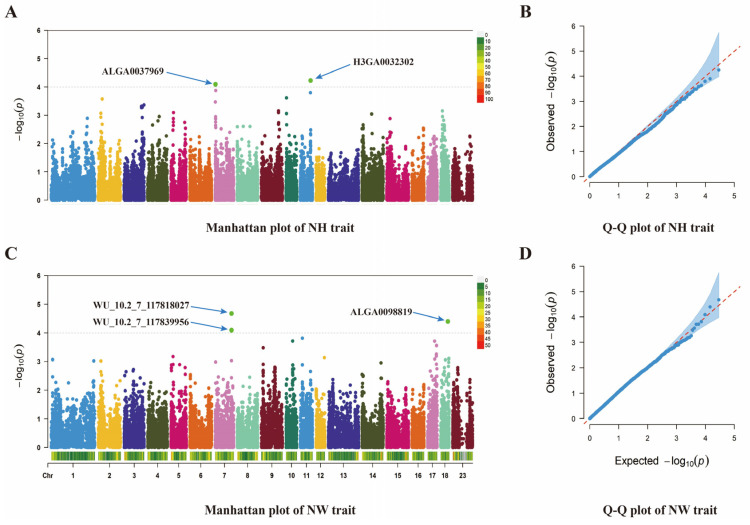
Manhattan plot and Q-Q plot based on two reproductive traits under combined parities. (**A**) Manhattan plot of the NH trait, displaying the genomic positions of SNPs on the *x*-axis and their corresponding −log10(*p*-values) on the *y*-axis. Significant SNPs are indicated by green dots. (**B**) Q-Q plot of the NH trait, illustrating the relationship between observed and expected −log10(*p*-values). The blue area represents the confidence interval, while the red dashed line represents the expected values under the null hypothesis. (**C**) Manhattan plot of the NW trait, displaying the genomic positions of SNPs on the *x*-axis and their corresponding −log10(*p*-values) on the *y*-axis. Significant SNPs are indicated by green dots. (**D**) Q-Q plot of the NW trait, illustrating the relationship between observed and expected −log10(*p*-values). The blue area represents the confidence interval, while the red dashed line represents the expected values under the null hypothesis. Note: Green dots in the Manhattan plot indicate significant SNPs. The horizontal axis represents the position of the marker on the pig chromosome, while the vertical axis represents the $-\log_{10}$(*p*-value) of the marker–trait association. The red, dashed line indicates the significance threshold criteria set in this study. The quantile–quantile (Q-Q) plot shows the relationship between the expected and the observed *p*-values. The horizontal axis represents the negative logarithm of the expected *p*-value, while the vertical axis represents the negative logarithm of the observed *p*-value for each SNP. The red, dashed line represents the expected relationship under the null hypothesis.

After the GWAS analysis of 385 valid individuals and 35,795 valid SNP markers, all of the parity data were firstly merged and analyzed, and a total of five SNP loci were screened for significant associations with the reproductive traits (Table 2), of which, ALGA0098819 was located in the QTL region that had been previously reported [4] and ALGA0037969, H3GA0032302, WU_10.2_7_117818027, and WU_10.2_7_117839956 had not been documented. In this study, five SNPs were found to be significantly correlated with the NH and NW traits in Large White pigs. Among them, the molecular markers ALGA0037969 and H3GA0032302 were found to be significantly correlated with NH traits. The three significant SNP loci associated with NW were ALGA0098819, WU_10.2_7_117818027, and WU_10.2_7_117839956. The quantile–quantile plots in Figure 3B,D show that the expected values in the initial portion closely matched the observed values, while the middle and latter portions gradually exhibited upward skewing. This suggests that the model effectively controls false positives and ensures the reliability of the results.

In Figure 3A, the Manhattan plot shows that the NH trait of 385 Large White pigs is significantly associated with two SNPs, ALGA0037969 and H3GA0032302, on 19 chromosomes, located on chromosomes 7 and 11, respectively. No candidate genes were annotated at these two SNPs. A total of 16 candidate genes were annotated within a range of 1 Mb above and below the ALGA0037969 locus, and 6 candidate genes were annotated within a range of 1 Mb above and below the H3GA0032302 locus (Table 2). Three significant SNPs for NW were identified at the loci ALGA0098819, WU_10.2_7_117818027, and WU_10.2_7_117839956. At the locus ALGA0098819, only the *BLVRA* gene was annotated, and a total of 16 genes were annotated within a 1Mb range above and below this SNP position (Table 2). No candidate genes were annotated directly at the SNPs WU_10.2_7_117818027 and WU_10.2_7_117839956, but six significant candidate genes were identified within a 1Mb range upstream and downstream of these SNPs: *BDKRB2*, *AK7*, *PAPOLA*, *VRK1*, *ATG2B*, and *GSKIP* (Table 2).

After the GWAS analysis of different parities (first to fourth parity) separately, differences in the significant SNPs associated with reproductive traits were found between the parities. The differences in these significant loci may reflect the variability in genetic mechanisms across litter sizes. In order to further explore the biological significance of these significant loci, a functional annotation of the significant loci in each parity was performed in this study.

**Figure 4:**
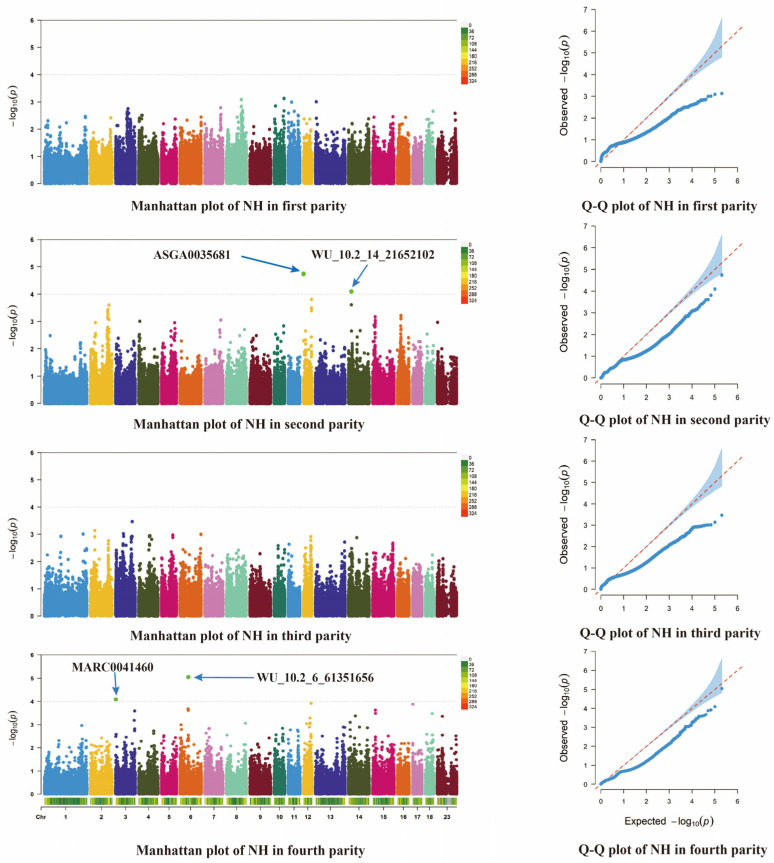
Manhattan plot and quantile–quantile plot based on different parity NH traits. The Manhattan plots (**left**) display the genomic positions of SNPs (*x*-axis) and their corresponding −log10(*p*-values) (*y*-axis). The Q-Q plots (**right**) show the relationship between observed and expected −log10(*p*-values). In the Q-Q plots, the blue area represents the confidence interval under the null hypothesis, while the red dashed line represents the expected values under perfect correlation between observed and expected *p*-values.

**Figure 5:**
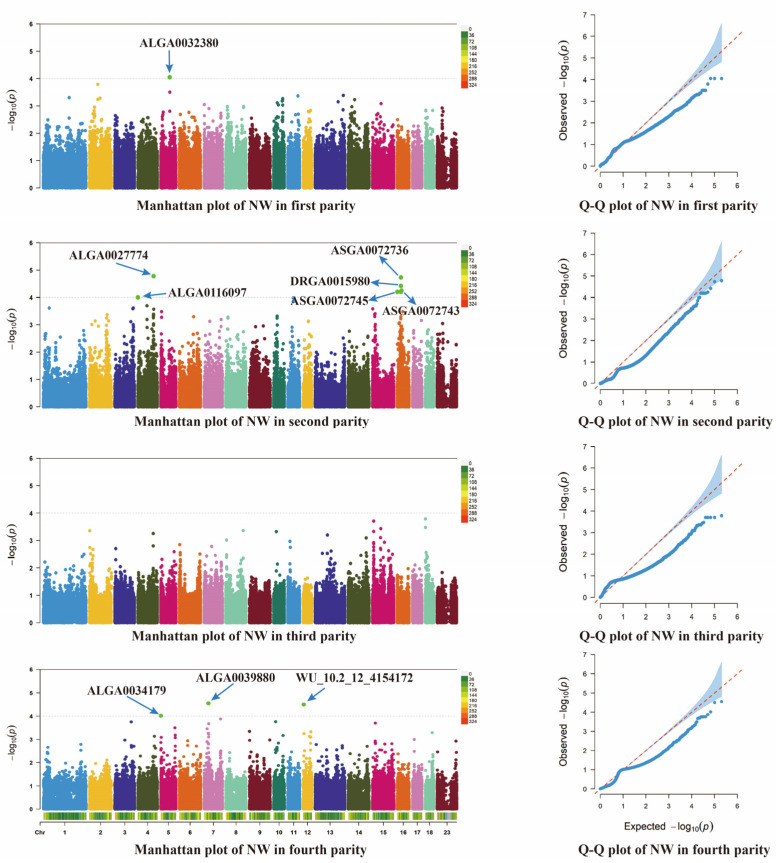
Manhattan plot and quantile–quantile plot based on different parity NW traits. The Manhattan plots (**left**) display the genomic positions of SNPs (*x*-axis) and their corresponding −log10(*p*-values) (*y*-axis). The Q-Q plots (**right**) show the relationship between observed and expected −log10(*p*-values). In the Q-Q plots, the blue area represents the confidence interval under the null hypothesis, while the red dashed line represents the expected values under perfect correlation between observed and expected *p*-values.

Figure 4 and Figure 5 show the results of the genome-wide association analysis (GWAS) for the NH and NW traits in the different parities; for the first parity, no significant locus was screened for the NH trait. However, one significant locus, ALGA0032380, was screened for the NW trait, and the closest candidate gene to this locus was annotated as *PRMT8*. In the second parity, two significant loci were identified for NH trait screening, located on chromosomes 12 and 14, respectively. The candidate genes closest to these loci were *MAP2K4* and *EXT1*, respectively. On the NW trait, six significant loci were screened, namely ALGA0116097 (located on chromosome 4), ASGA0072736 (chromosome 16), ALGA0027774 (chromosome 4), DRGA0015980, and ASGA0072745 and ASGA0072743 (both located on chromosome 16). The annotated candidate genes were *MED30*, *U2*, *OLFM3*, *MROH2B*, and ASGA0072745 and ASGA0072743, both of which were annotated with the *PLCXD3* gene. For the third generation, no significant loci were screened in that generation. For the fourth generation, two significant loci were screened for the NH trait, MARC0041460 (chromosome 3) and WU_10.2_6_61_351656 (chromosome 6), and the closest candidate genes annotated to these two significant loci were *RNASEH1* and *PLCXD3*. For the NW trait, three significant loci were screened, namely ALGA0034179 (chromosome 5), ALGA0039880 (chromosome 7), and WU_10.2_12_4_154172 (chromosome 12). The candidate genes annotated to these significant loci were *PYM1*, *ANKS1A*, and *SEPTIN9*, respectively (Table 3).

### 3.2. Functional Enrichment Analysis of Candidate Genes Reveals Key Biological Processes and Signaling Pathways

**Figure 6:**
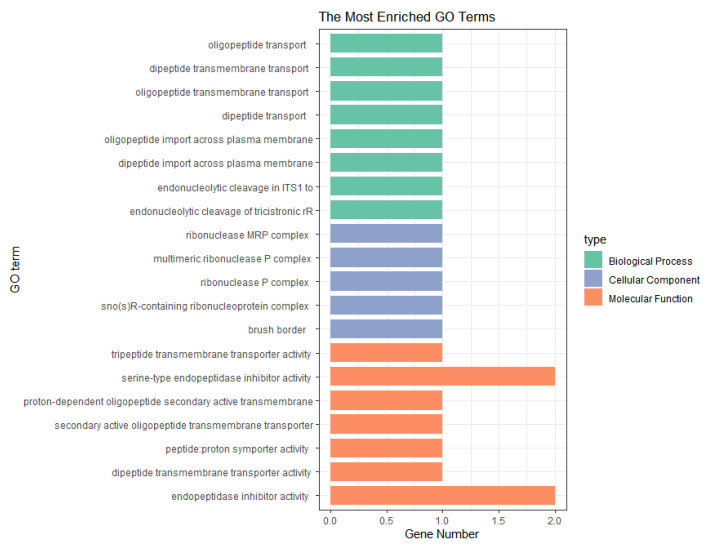
The GO enrichment analysis of significant SNPs for the NH trait with combined parities.

Candidate genes that were screened based on all of the parities were analyzed for GO and KEGG enrichment using the clusterProfiler package (4.10.1) [20] in conjunction with the gene ontology database. For the NH trait, the enrichment analysis showed that the *RPP40*, *SLC15A1*, *SERPINB1*, *SERPINB9*, and *ECI2* genes were significantly enriched in RNA processing, endonuclease activity, fatty acid binding, and other aspects (*p* < 0.05) (Figure 6). The KEGG pathway analysis identified two significant pathways, namely Salmonella infection and intercellular junction, involving the *RIPK1* and *TUBB2B* genes (Table 4).

**Figure 7:**
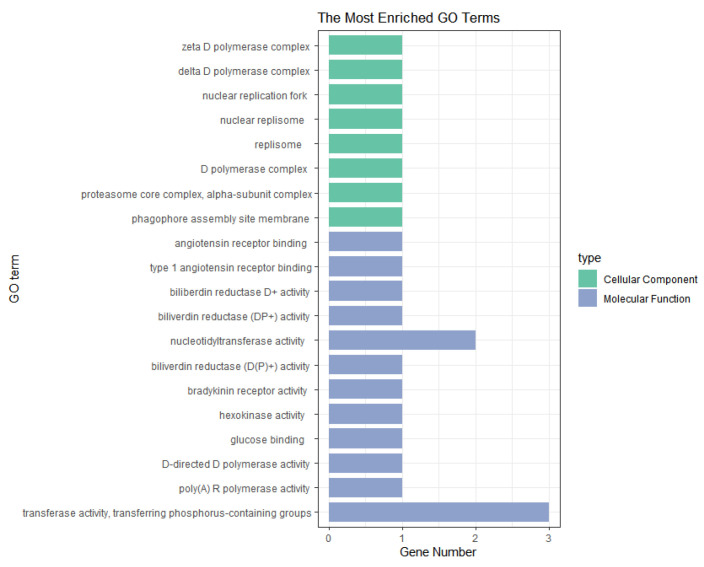
The GO enrichment analysis of significant SNPs for the NW trait with combined parities.

For the NW traits, the GO analysis screened for significantly enriched terms (*p* < 0.05) for genes, such as *POLD2*, *PSMA2*, *BDKRB2*, *BLVRA*, *GCK*, *POLM*, and *PAPOLA*, which are involved in biological functions such as DNA polymerase complexes, cholecystokinin reductase activity, and the binding of regulatory subunits of protein kinase A and encompass processes such as metabolism and energy homeostasis (Figure 7). No significant pathways were screened in the KEGG analysis.

We performed GO and KEGG enrichment analyses on candidate genes selected from different parities using the clusterProfiler package [20] combined with the gene ontology database (Table 5). For the NW trait in the first parity, the GO functional enrichment analysis showed significant enrichment for GO:0018216 (peptidyl-arginine). This gene, *PRMT8*, is associated with protein modification and, in particular, plays a role in methylation. No significant GO entries were enriched for the NH trait for the second parity, whereas the GO enrichment analysis for the NW trait showed significant enrichment for multiple GO entries, including GO:0060261 (the RNA polymerase II-mediated positive regulation of transcription initiation), GO:0006352 (DNA template transcription initiation), GO:0019827 (stem cell population maintenance), and GO:0008081 (phosphodiesterase hydrolase activity). These entries suggest that the two genes, *MED30* and *PLCXD3*, may play important roles in transcriptional regulation, stem cell population maintenance, and phosphatase activity. In the fourth parity of the NH trait, the GO functional enrichment analysis showed GO:0043137 (RNA primer removal) and GO:0042578 (phosphate hydrolase activity), suggesting that the *RNASEH1* and *PLCXD3* genes may play an important regulatory role in the process of DNA replication and phosphate hydrolysis. The GO enrichment analysis of NW traits in the fourth parity indicated multiple significant GO entries, including GO:1903259 (the dissociation of the exon–exon junction complex), GO:0032984 (the dissociation of the protein-containing complex), GO:0022411 (the dissociation of cellular components), and GO:0005525 (GTP-binding), suggesting that the *PYM1* and *SEPTIN9* genes may be associated with cytoskeleton dynamics, signaling, and GTP-binding processes. The KEGG analysis was conducted on different parities, and no significant pathways were identified in the candidate gene sets of the first, second, and fourth parities.

### 3.3. Comparison of Phenotypic Prediction Performance Across Different Models

**Figure 8:**
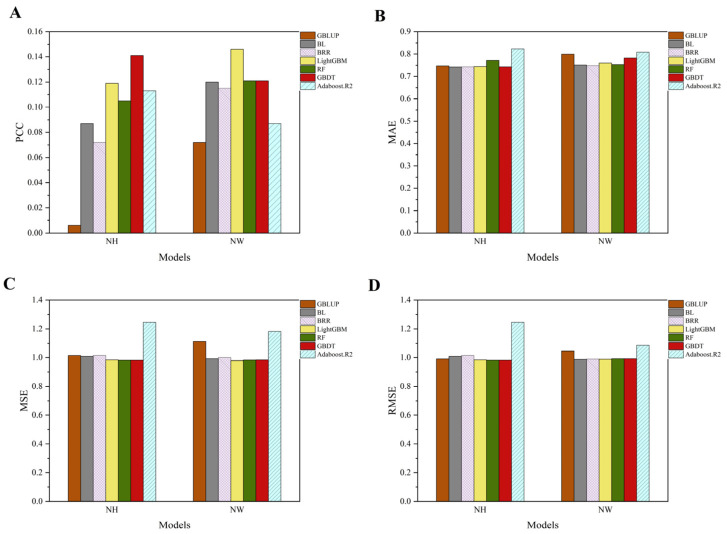
Comparison of model performance using the PCA method for predicting two traits. (**A**) Correlation coefficient (PCC) of different models (GBLUP, BL, BRR, LightGBM, RF, GBDT, and AdaBoost.R2) for NH and NW traits. (**B**) Mean absolute error (MAE) of the models for NH and NW traits. (**C**) Mean squared error (MSE) of the models for NH and NW traits. (**D**) Root mean squared error (RMSE) of the models for NH and NW traits.

In the study of the phenotype prediction of reproductive traits (NH and NW traits) of 385 Large White pigs, different models were used and different proportions of SNP numbers were randomly selected for the prediction (Table 6 and Table 7). From the prediction results, it can be seen that there are differences in the performance of different models on the SNP data with different proportions and after PCA processing (Figure 8). Regarding using the PCA method to predict two traits (NH and NW), the optimal hyperparameters for each machine learning model are shown in Table 8.

In the prediction of NH traits, the PCC results of the models differed under different proportions of SNP data. For the 20% SNP proportion, the GBLUP model had a weak PCC value of −0.116, while the GBDT model had the best PCC value of 0.131. For the 50% SNP proportion, the PCC value of GBLUP model decreased to −0.129, the lowest among all the models, and the PCC value of the LightGBM model was 0.057, which was a relatively good performance. With the percentage of SNPs increased to 80%, the PCC value of the GBLUP model improved but remained negative (−0.119), at which point, the PCC value of the Adaboost.R2 model was 0.059, which was a relatively better performance. When using all of the SNP data, the GBLUP model had a PCC value of −0.12, while the Adaboost.R2 model had a better performance, with a PCC value of 0.071. After PCA processing, the PCC value of GBLUP was improved to 0.011, and the PCC value of GBDT model reached 0.141, which made it the best-performing model among all of the models. In terms of MAE, the MAE values of the GBLUP, BL, and BRR models were relatively stable (about 0.768), while LightGBM has the lowest MAE (0.744) after PCA treatment. In terms of MSE and RMSE metrics, the GBDT and RF models had the smallest errors after PCA treatment (MSE of 0.982), whereas the Adaboost R2 model had the highest MSE and RMSE values for all the SNP proportions, especially for all of the SNP data, with an MSE of 1.546 and an RMSE of 1.243.

In the prediction of NW traits, the results of the PCC, MAE, MSE, and RMSE of each model under different proportions of SNP data were as follows: in terms of PCC, the GBLUP model had negative PCC values under different proportions of SNP data, and the PCC at the 20% SNP proportion was −0.111, which was a poor performance, while the Adaboost.R2 model had a PCC value of 0.064, the best performance. As the SNP proportion increased to 50%, the PCC value of the GBLUP model further decreased to −0.119, which was the lowest among all the models, while the PCC value of the Adaboost.R2 model was 0.047, which was the highest value at this proportion. At the 80% SNP ratio, the PCC value of the GBLUP model decreased to −0.121, and the RF model had the best performance, with a PCC value of 0.047. Under all of the SNP data, the GBLUP model had a PCC value of −0.114 and the Adaboost.R2 model had a PCC value of 0.062, which was the best performance. After PCA processing, the PCC of the GBLUP model improved and raised to 0.072, while the PCC of the LightGBM model had a value of 0.146, making it the best performer among all of the models. In terms of MAE, the GBLUP, BL, and BRR models had more stable MAE values, which were about 0.777 or so for each SNP ratio. In terms of MSE and RMSE, the traditional models, such as GBLUP, BL, and BRR, had relatively high errors, especially under all of the SNP data; the GBLUP model had an MSE of 1.066 and an RMSE of 1.027. After PCA treatment, the LightGBM model performed better in terms of the MSE and RMSE, which were 0.979 and 0.989, respectively. In contrast, the Adaboost.R2 model had the highest MSE and RMSE for all of the SNP ratios; in particular, after PCA treatment, the MSE reached 1.182 and the RMSE was 1.087.

## 4. Discussion

In pig breeding, selecting superior individuals is a key strategy to enhance economic efficiency. Reproductive traits, such as the number of healthy litters and the number of weaned heads in Large White pigs, are essential indicators of sow productivity. However, traditional phenotypic selection methods have limitations, as these traits are controlled by microefficient polygenes and have low heritability. Therefore, combining gene identification with phenotypic selection, along with the use of genomic selection (GS) models, allows for a deeper exploration of the genetic mechanisms underlying reproductive traits, facilitates an understanding of their complex genetic networks, and improves the efficiency of phenotype prediction. This approach is crucial for enhancing breeding efficiency and guiding the development of the pig breeding industry.

In this study, after combining all of the parities, genes closely associated with reproductive traits in Large White pigs, including *BLVRA*, *STK17A*, *PSMA2*, and *C7orf25*, were screened near the significant SNP locus ALGA0098819. Additionally, in Wang’s study [4], the QTL region, where this SNP locus is located, was found to be associated with litter yield and to be in proximity to several related genes. ALGA0037969, H3GA0032302, WU_10.2_7_117818027, and WU_10.2_7_117839956 have not been previously documented as molecular markers associated with NH and NW in Large White pigs. These findings will provide a basis for subsequent genetic studies on Large White pigs.

GWASs have been widely used to screen genes associated with porcine reproductive traits. Zhao [31] identified eight candidate genes (including *INHBA*, *LEPR*, and *HMX1*) associated with litter size, providing insights into the genetic architecture of reproductive traits in pigs. Other studies have also revealed the genetic basis of reproductive traits in different pig breeds, including the significant effect of the *DPF3* and *NRP1* genes on nipple-number-related traits [32], as well as potential candidate genes associated with steroid hormone receptor activity in Danish Large White pigs [33]. Collectively, these studies have enhanced the understanding of the genetic and biological mechanisms underlying reproductive traits in pigs.

The candidate genes related to reproductive traits were assessed when all the parities were merged, and it was found that *SERPINB9*, *IPO5*, *BLVRA*, *AK7*, *BDKRB2*, and *VRK1* were closely related to reproductive traits [33,34,35,36,37,38].

In this study, the *RPP40*, *ECI2*, *SLC15A1*, *SERPINB1*, *RIPK1*, and *TUBB2B* genes demonstrated their respective important regulatory roles in different biological processes. *RPP40* [34] regulates immune response and the ecto-matrix in tumor microenvironments, suggesting its role in cell proliferation and homeostasis. It is reported in the literature that the *ECI2* gene is involved in fatty acid metabolism and significantly correlates with pork texture traits [35]. *SLC15A1* affects intestinal health and immune response by regulating dipeptide and tripeptide transport [36]. *SERPINB1* acts as a protease inhibitor to prevent inflammation-induced tissue damage and modulates the immune system [37]. Previous studies also suggest that the *RIPK1* gene plays a key role in cell death and necrotic apoptosis and is particularly closely associated with cell growth and development, which may indirectly affect body size and fat deposition [38]. The *TUBB2B* gene is closely associated with neuronal migration and cortical development, and mutations in it may lead to cortical malformations such as anencephaly and multi-tiny, gyrus-like dysplasia, which can affect neurological function. The *TUBB2B* gene has been implicated in the development of neuronal migration and the development of the cortex [39].

Despite the important roles of these genes in metabolism, immune regulation, and developmental processes, no studies have clearly shown that they are directly related to reproductive traits in pigs. However, these genes may indirectly affect reproductive health through the regulation of metabolic and developmental processes, particularly in sow health management and embryo development, and future studies could further explore the potential impact of these genes in porcine reproductive traits.

In this study, the *PSMA2*, *GCK*, *POLD2*, *POLM*, and *PAPOLA* genes exhibited a variety of biological functions, and their important roles in several fields have been widely reported in the literature. For example, the association between *PSMA2* and cancer and type 2 diabetes has been demonstrated, suggesting its critical position in tumorigenesis [40]. Studies have shown that *GCK* genes play a particularly prominent role in glucose metabolism and lipid synthesis, especially showing their potential impact in feed efficiency regulation [41]. The *POLD2* gene plays a central role in DNA replication and repair, safeguarding normal cellular function by maintaining genome stability [42]. The *POLM* gene is involved in the non-homologous end-joining repair mechanism, ensuring rapid repair after DNA damage, and the literature supports its diverse functions in the immune system [43]. The ability of the *PAPOLA* gene to regulate sperm quality has been experimentally verified, especially in the RNA tailing modification [44].

Although these genes have important functions in metabolism, DNA repair, and immune regulation, there is no clear evidence that they are directly associated with reproductive traits in pigs. However, the *GCK* and *AEBP1* genes may have indirect effects on the reproductive health of sows by regulating lipid metabolism and energy homeostasis, while the *PAPOLA* gene may have a role in reproductive performance by affecting sperm quality. Therefore, further studies on the potential effects of these genes in porcine reproductive traits are necessary in the future to expand the understanding of their functions.

Porcine circovirus type 2 (PCV2) is the primary cause of postweaning multisystemic wasting syndrome (PMWS) in pigs, and it can prevent porcine alveolar macrophages (PAMs) from undergoing apoptosis by upregulating *SERPINB9* expression. Additionally, the introduction of the host protein *IPO5* during PCV2 infection helps maintain cellular stability, inhibit virus production, and prevent protease degradation. This gene is related to the number of weaned heads and can be considered a candidate gene for this trait [45,46]. Bilirubin reductase (*BLVRA*) binds to dying sperm, and the enzyme loop involved in *BLVRA* activity acts as a reactive oxygen species (ROS) scavenger, protecting living epididymal sperm from harmful ROS released by dying cells. This protection from oxidative stress ensures high levels of expression in fertile sperm, which may be closely related to healthy piglet traits [47]. *AK7* and *BDKRB2* are associated with spermatogenesis and play roles in the maturation of spermatozoa in the seminiferous tubules and the acquisition of sperm viability in the epididymis. Additionally, BDKRB2 regulates the *AQP9* water channel in the epididymis of mice and the transport of particles in the vas deferens of humans and pigs [48]. *AK7* has been linked to infertility and spermatogenesis failure and may be associated with defects in tail formation, making it a potential molecular marker for the number of healthy litters among reproductive traits [49]. The role of *VRK1* in the early stages of spermatogenesis underscores its importance in the reproductive process [50].

In an analysis of GO functional annotation across parities, the *PLCXD3* gene was annotated in the NW trait in two parities (second and fourth). Studies on the *PLCXD3* gene have been reported in the literature to be mainly involved in phospholipid metabolism and cell signaling [51]. *PLCXD3* is a member of the phosphatidylinositol-specific phospholipase C family of enzymes, which are involved in the hydrolysis of membrane phospholipids and play a key role in a variety of cellular metabolic processes. *PLCXD3* expression has an important role in glucose sensing and insulin signaling in pancreatic islet β-cells, and its gene knockdown resulted in a significant decrease in insulin secretion, suggesting a possible association between this gene and metabolic diseases. In addition, *PLCXD3* plays an important role in processes related to signaling and cytoskeleton regulation, especially in signaling pathways related to cell proliferation and differentiation, and may play a role in the regulation of the reproductive system [52].

Regarding the *RNASEH1* gene, previous reports indicate that it is mainly involved in RNA primer removal during DNA replication and DNA repair, which is essential for maintaining genome stability [53]. *RNASEH1* is widely expressed in different cell types and is tightly associated with mitochondrial DNA replication and the maintenance of function. Studies have shown that mutations in this gene may lead to the development of mitochondria-associated diseases, suggesting its importance in metabolic regulation [54]. The *PYM1* gene is involved in the regulation of ribosomal function, which plays a role in mRNA de-capping and translation, which, in turn, affects protein synthesis. Although the specific mechanism in reproductive traits has not been fully elucidated, it may indirectly affect the reproductive system in the regulation of cell growth and metabolism [55]. *SEPTIN9* is a member of the Septin family of proteins [56], which play important roles in cell division, especially in cytoskeleton construction and regulation. *SEPTIN9* has been widely studied in cancer, and the overexpression of *SEPTIN9*, especially in breast cancer, suggests that it may have an important effect in cell proliferation and division. Therefore, *SEPTIN9* may indirectly affect reproductive traits by influencing cell proliferation.

To summarize, although the specific mechanisms of *PRMT8* [55,57], *MED30* [58], *PLCXD3*, *RNASEH1*, *PYM1*, and *SEPTIN9* [59] genes in reproductive traits have not been fully clarified, it has been demonstrated that they play important roles in fundamental processes such as cell signaling, metabolic regulation, DNA repair, and cell division. This provides a theoretical basis for their potential functions in complex traits such as reproductive traits.

In the combined parity analysis, we applied a strategy of selecting candidate genes within a 1 Mb region both upstream and downstream of each significant SNP. This approach identified five significant SNPs and resulted in the annotation of 45 candidate genes. The rationale for employing this broader selection range was to capture more general and widespread genetic effects, particularly those that may persist across multiple parities. By integrating the data across all the parities, we enhanced the statistical power, enabling the detection of gene regulatory networks that function across the various parities. The use of an expanded candidate gene selection range is a common strategy in studies of complex traits regulated by multiple genes, as it increases the likelihood of detecting polygenic effects [60,61].

In contrast, for the by-parity analysis, we selected the gene closest to each significant SNP. This approach yielded 13 significant SNPs and 12 candidate genes. The objective of the by-parity analysis was to pinpoint gene effects that are most prominent and specific to each parity. By employing a more precise selection approach, we aimed to improve the accuracy of detecting associations between specific genes and target traits, particularly considering the varying physiological conditions across parities. This strategy enables the detection of genes with potentially stronger effects within specific parities [62,63].

In this study, based on PCC values, even the best models exhibited a limited predictive accuracy. Several factors likely contribute to the observed low PCC values. First, traits with low heritability are generally challenging to predict genomically. Heritability reflects the genotype’s contribution to the phenotype; with low heritability, the genotype exerts a minimal effect on trait expression, making it difficult for the model to capture these subtle effects, which, in turn, lowers the prediction accuracy and PCC values. Prediction accuracy in genomic selection significantly declines with lower heritability [64]. Secondly, limited data volume is another critical factor contributing to low PCC values. In predicting complex traits, increasing the SNP marker density and sample size can enhance the model’s predictive power. Expanding data volume is a key factor in improving predictive performance [65]. Larger datasets provide the improved resolution of genotype–phenotype relationships, particularly for complex trait predictions. Third, environmental effects also play a significant role in complex trait prediction. Reproductive traits, such as litter size and the number of weaned piglets, are strongly influenced by environmental factors. Even with model adjustments for some environmental effects, unmeasured or unaccounted environmental factors can still negatively impact predictive accuracy [66]. Additionally, traits like NH and NW are often co-regulated by multiple small-effect genes, adding complexity to their prediction. The polygenic regulation of complex traits and the involvement of small-effect genes challenge the model’s ability to accurately capture these effects, thus affecting the prediction outcomes [67]. This complexity further explains the low PCC values, particularly in traits with polygenic regulation. To enhance the model’s predictive accuracy, future studies may consider the following: increasing the data volume, particularly with samples covering diverse environmental conditions, to improve the model’s capacity for capturing gene–environment interactions; using more precise phenotypic data to reduce the measurement error and better capture trait variation; and incorporating advanced models, such as deep learning or other machine learning approaches, to more effectively capture gene effects in complex traits.

In this study, the PCA method was used for the dimensionality reduction processing of the genotype data, which enhanced the prediction ability of the machine learning models. PCA, as a classical dimensionality reduction method, can effectively reduce the redundant information in high-dimensional data while maintaining the key features in the data, so that the prediction performance of the model can be improved. This enhancement is mainly reflected in the following aspects:

Firstly, PCA effectively reduces the noise and redundant information present in high-dimensional gene data by reducing the feature dimension. Genetic data usually has high dimensions and strong correlations between features. Directly using raw data increases the complexity of the model and can easily lead to overfitting. PCA transforms the original features into irrelevant principal components, preserves the maximum amount of information in the data, reduces useless or redundant information, and, thus, makes machine learning models more generalizable during training. This is also supported in the literature, as dimensionality reduction can significantly improve the performance of the model on high-dimensional data [67]. Second, PCA’s ability to handle multicollinearity significantly enhances model stability and accuracy. Multicollinearity is prevalent in high-dimensional data, and, especially, linear models (e.g., GBLUP, BL, and BRR) are sensitive to multicollinearity, which can easily lead to unstable model estimation. PCA eliminates the multicollinearity problem by combining highly correlated features into independent principal components, which improves the prediction performance of these models on complex genetic datasets [68].

In addition, PCA reduces the data dimensionality, allowing machine learning models to improve their computational efficiency and convergence speed. For machine learning models like GBDT and LightGBM, more highly dimensional data often increases the computational time and complexity of the model. PCA reduces the model training time by compressing the feature space and ensures an improved prediction performance while reducing the computational burden, which enhances the application efficiency of the model [67].

Furthermore, the findings suggest that hyperparameter tuning is critical for optimizing the performance of machine learning models. The performance of different models depends significantly on their hyperparameter settings (Table 8). Default hyperparameter configurations typically do not fully exploit the models’ potential, while tuning the hyperparameters through optimization algorithms (e.g., grid search, random search, or Bayesian optimization) can significantly enhance their predictive power. This optimization process enables the model to better adapt to data complexity and accurately capture nonlinear patterns and feature interactions.

In breeding practice, the ability of models to accurately predict outcomes directly impacts the selection of superior individuals and the development of breeding strategies. Therefore, hyperparameter adjustment is not only a method to improve model performance but also a key step in achieving breeding goals. By optimizing models to improve their prediction accuracy, researchers can more reliably identify individuals with superior reproductive traits, thereby guiding practical breeding decisions. This process ultimately enhances breeding efficiency and economic outcomes, providing more precise support for breeding decisions.

Although this study has made significant progress, the size and diversity of the dataset needs to be expanded to enhance the model’s generalization ability. Future research should incorporate more environmental variables to validate the model’s adaptability under varying conditions. Additionally, the research results will be applied to actual production settings to test their effectiveness in real-world environments, providing further validation and directions for optimization.

## 5. Conclusions

In this study, we conducted a genome-wide association study (GWAS) on the number of healthy piglets (NH) and the number of weaned piglets (NW) in a cohort of 385 Large White pigs, using both combined-parity and by-parity data. In the combined-parity analysis, we identified five SNPs significantly associated with reproductive traits and annotated 45 candidate genes located within a 1Mb region upstream and downstream of these SNPs. The GO and KEGG enrichment analyses included 14 of these genes. A literature review revealed that genes, including *SERPINB9*, *IPO5*, *BLVRA*, *AK7*, *BDKRB2*, and *VRK1*, have previously been linked to reproductive traits. In the by-parity analysis, we identified 13 significant SNPs and annotated 12 candidate genes by selecting the genes closest to each significant SNP. The GO and KEGG analyses included six of these genes. Although the precise mechanisms by which *PRMT8*, *MED30*, *PLCXD3*, *RNASEH1*, *PYM1*, and *SEPTIN9* influence reproductive traits remain unclear, existing studies suggest that these genes are involved in essential biological processes, including cell signaling, metabolic regulation, DNA repair, and cell division, which may contribute to their roles in reproductive traits. We further compared the phenotypic prediction performance of several machine learning models, including LightGBM, RF, GBDT, and Adaboost.R2, with traditional genomic prediction models, such as GBLUP, BRR, and BL. All of the models showed relatively low PCC values; even in the best-performing models, the predictive accuracy remained modest. However, all of the models showed a performance improvement after applying a principal component analysis (PCA).

## Figures and Tables

**Table 1:** Descriptive statistics of reproductive traits in Large White pigs.

Trait	Mean	Standard Deviation	Coefficient of Variation	Minimum	Maximum
NH	13.34	2.68	20.28%	4	21
NW	11.38	1.72	15.16%	4	15

Note: NH and NW represent the number of healthy litters and the number of weaned heads of Large White pigs, respectively. This notation is consistent throughout the text.

**Table 2:** Candidate genes within 1Mb of significant SNP loci.

Trait	Chr	*p* Value	Starting Physical Position/bp	Terminate Physical Position/bp	Candidate Gene
**ALGA0037969**					
NH	7	7.7 × 10^−5^	1,684,215	1,692,980	*SERPINB1*
			1,792,876	1,805,903	*NQO2*
			1,841,239	1,879,765	*RIPK1*
			1,882,720	1,909,854	*BPHL*
			1,656,984	1,667,879	*WRNIP1*
			1,721,383	1,735,074	*SERPINB9*
			1,910,269	1,914,668	*TUBB2A*
			1,951,407	1,956,119	*TUBB2B*
			1,988,696	2,131,908	*SLC22A23*
			2,309,088	2,310,080	*FAM50B*
			2,382,495	2,408,674	*PRPF4B*
			2,421,054	2,446,319	*ECI2*
			2,231,293	2,248,863	*PXDC1*
			2,846,090	2,966,240	*CDYL*
			2,987,271	3,005,271	*RPP40*
			1,752,689	1,765,107	*SERPINB6*
**H3GA0032302**					
NH	11	5.7 × 10^−5^	66,481,923	66,641,651	*MBNL2*
			66,679,253	66,720,045	*RAP2A*
			67,004,109	67,059,988	*IPO5*
			67,144,645	67,457,543	*FARP1*
			67,458,352	67,615,842	*STK24*
			67,652,193	67,710,933	*SLC15A1*
**ALGA0098819**					
NW	18	4.0 × 10^−5^	51,131,584	51,185,435	*BLVRA*
			51,322,240	51,362,177	*STK17A*
			51,909,473	51,921,238	*PSMA2*
			51,928,145	51,928,145	*C7orf25*
			50,609,418	50,684,964	*OGDH*
			50,726,922	50,757,649	*NPC1L1*
			50,699,470	50,702,704	*TMED4*
			50,705,632	50,715,033	*DDX56*
			50,759,221	50,830,332	*NUDCD3*
			50,861,466	50,956,225	*CAMK2B*
			50,979,167	51,024,494	*GCK*
			50,960,113	50,971,029	*YKT6*
			51,046,899	51,053,854	*AEBP1*
			51,038,358	51,046,779	*POLD2*
			51,079,503	51,088,781	*POLM*
			51,387,836	51,802,945	*HECW1*
			52,404,072	52,697,900	*GLI3*
**WU_10.2_7_117818027**					
NW	7	8.1 × 10^−5^	117,438,622	117,472,005	*BDKRB2*
			117,609,791	117,667,247	*AK7*
			117,676,083	117,740,348	*PAPOLA*
**WU_10.2_7_117839956**					
NW	7	2.1 × 10^−5^	117,942,929	118,025,991	*VRK1*
			117,506,298	117,586,651	*ATG2B*
			117,586,742	117,607,575	*GSKIP*

**Table 3:** Significant SNP loci and corresponding candidate genes in the GWAS, based on different parity.

Trait	SNP	Chr	Position/bp	*p* Value	Nearest Gene	Location ^1^
**First Parity**						
NW	ALGA0032380	5	66,707,514	8.926 × 10^−5^	*PRMT8*	within
**Second Parity**						
NH	ASGA0035681	12	56,494,112	9.981 × 10^−5^	*MAP2K4*	within
NH	WU_10.2_14_21652102	14	162,537,498	8.056 × 10^−5^	*EXTI*	89,715
NW	ALGA0116097	4	21,319,382	9.981 × 10^−5^	*MED30*	90,972
NW	ASGA0072736	16	24,798,218	6.289 × 10^−5^	*U2*	17,042
NW	ALGA0027774	4	116,167,774	1.650 × 10^−5^	*OLFM3*	49,569
NW	DRGA0015980	16	25,960,781	6.289 × 10^−5^	*MROH2B*	within
NW	ASGA0072745	16	26,154,317	6.289 × 10^−5^	*PLCXD3*	2001
NW	ASGA0072743	16	26,247,215	6.289 × 10^−5^	*PLCXD3*	within
**Fourth Parity**						
NH	MARC0041460	3	131,287,295	8.089 × 10^−5^	*RNASEH1*	5365
NH	WU_10.2_6_61351656	6	61,351,656	8.902 × 10^−6^	*PLCXD3*	within
NW	ALGA0034179	5	21,319,382	9.687 × 10^−5^	*PYM1*	within
NW	ALGA0039880	7	30,978,428	2.793 × 10^−5^	*ANKS1A*	within
NW	WU_10.2_12_4154172	12	4,154,172	3.152 × 10^−5^	*SEPTIN9*	48,323

^1^ Locations represent the distance between significant SNPs.

**Table 4:** KEGG pathway information of significant SNP candidate genes in combined parities.

Trait	Pathway	Description	Candidate Gene	γ Value
NH	ssc05132	Salmonella infection	*TUBB2B/RIPK1*	0.028
	ssc04540	Gap junction	*TUBB2B*	0.032

**Table 5:** Results of GO functional annotation of candidate genes for two trait significant SNP loci in different parities.

Parity	Trait	GO Terms	Gene Name
**First parity**	NW	GO: 0018216, peptidyl-arginine methylation GO: 0006479, protein methylation GO: 0018193, peptidyl-amino acid modification	*PRMT8*
**Second parity**	NH	No significant entries are enriched	NULL
**Fourth parity**	NW	GO: 0060261, positive regulation of transcription initiation by RNA polymerase II GO: 0006352, DNA-templated transcription initiation GO: 0019827, stem cell population maintenance GO: 0008081, phosphoric diester hydrolase activity	*MED30/PLCXD3*
	NH	GO: 0043137, DNA replication, removal of RNA primer GO: 0042578, phosphoric ester hydrolase activity	*RNASEH1/PLCXD3*
	NW	GO: 1903259, exon-exon junction complex disassembly GO: 0032984, protein-containing complex disassembly GO: 0022411, cellular component disassembly GO: 0005525, GTP binding	*PYM1/SEPTIN9*

**Table 6:** The predictive performance of the different models using various proportions of SNP data and PCA methods for the NH trait.

Evaluation Indicators	Features	Models						
		GBLUP	BL	BRR	LightGBM	RF	GBDT	Adaboost.R2
**PCC**	20%	−0.116	−0.086	−0.092	−0.056	0.096	0.131	0.0233
	50%	−0.129	−0.103	−0.097	0.057	−0.035	−0.057	0.021
	80%	−0.119	−0.098	−0.095	0.013	−0.004	0.014	0.059
	All	−0.12	−0.095	−0.089	0.021	−0.048	−0.009	0.071
	PCA	0.011	0.087	0.072	0.119	0.105	0.141	0.113
**MAE**	20%	0.778	0.755	0.773	0.748	0.757	0.752	0.742
	50%	0.778	0.771	0.765	0.801	0.779	0.827	0.742
	80%	0.777	0.777	0.772	0.808	0.78	0.811	0.784
	All	0.777	0.776	0.79	0.807	0.774	0.822	0.962
	PCA	0.747	0.742	0.743	0.744	0.771	0.743	0.823
**MSE**	20%	1.07	1.026	1.059	1.014	1.019	0.996	1.002
	50%	1.07	1.064	1.044	1.121	1.071	1.195	1
	80%	1.068	1.067	1.059	1.149	1.076	1.151	1.111
	All	1.068	1.07	1.061	1.138	1.069	1.175	1.546
	PCA	1.014	1.008	1.015	0.984	0.982	0.982	1.245
**RMSE**	20%	1.029	1.005	1.022	1.007	1.01	0.998	1.001
	50%	1.029	1.023	1.014	1.059	1.035	1.093	1
	80%	1.028	1.025	1.021	1.072	1.038	1.073	1.054
	All	1.028	1.026	1.022	1.067	1.034	1.084	1.243
	PCA	1.006	0.996	0.999	0.992	0.991	0.991	1.116

**Table 7:** The predictive performance of the different models using various proportions of SNP data and PCA methods for the NW trait.

Evaluation Indicators	Features	Models						
		GBLUP	BL	BRR	LightGBM	RF	GBDT	Adaboost.R2
**PCC**	20%	−0.111	0.045	0.047	−0.016	0.052	0.012	0.064
	50%	−0.119	0.041	0.029	0.011	0.108	0.037	0.047
	80%	−0.121	0.04	0.029	0.001	0.047	−0.013	0.032
	All	−0.114	0.043	0.036	0.053	0.044	0.016	0.062
	PCA	0.072	0.12	0.115	0.146	0.121	0.121	0.087
**MAE**	20%	0.778	0.786	0.764	0.831	0.78	0.817	0.751
	50%	0.778	0.763	0.765	0.821	0.766	0.815	1.084
	80%	0.778	0.767	0.764	0.833	0.771	0.829	0.767
	All	0.777	0.764	0.765	0.816	0.771	0.821	0.760
	PCA	0.799	0.751	0.748	0.76	0.753	0.782	0.808
**MSE**	20%	1.068	1.019	1.034	1.177	1.035	1.159	1.078
	50%	1.068	1.031	1.04	1.17	1.006	1.148	0.740
	80%	1.069	1.045	1.041	1.183	1.035	1.18	1.111
	All	1.066	1.043	1.041	1.141	1.035	1.156	1.096
	PCA	1.112	0.992	1	0.979	0.983	0.984	1.182
**RMSE**	20%	1.027	0.999	1.007	1.085	1.017	1.077	1.038
	50%	1.027	1.006	1.011	1.082	1.003	1.071	1.041
	80%	1.028	1.014	1.011	1.087	1.017	1.086	1.054
	All	1.027	1.012	1.011	1.068	1.017	1.075	1.047
	PCA	1.045	0.987	0.99	0.989	0.992	0.992	1.087

**Table 8:** The optimal hyperparameters for the machine learning models in predicting two traits using the PCA method.

Trait	Method	Optimal Hyperparameters
NH	LightGBM	learning_rate = 0.01, max_depth = 19, n_estimators = 25
	RF	max_depth = 6, n_estimators = 87
	GBDT	learning_rate = 0.05, max_depth = 8, n_estimators = 10
	Adaboost.R2	n_estimators = 50, learning_rate = 0.01
NW	LightGBM	learning_rate = 0.1, max_depth = 2, n_estimators = 4
	RF	max depth = 1, n_estimators = 17
	GBDT	learning_rate = 0.09, max_depth = 1, n_estimators = 16
	Adaboost.R2	n_estimators = 50, learning_rate = 0.01

## Data Availability

The original contributions presented in the study are included in the article, further inquiries can be directed to the corresponding author.
